# Installation of an Artificial Vegetating Island in Oligomesotrophic Lake Paro, Korea

**DOI:** 10.1155/2013/857670

**Published:** 2013-07-15

**Authors:** Eun-Young Seo, Oh-Byung Kwon, Seung-Ik Choi, Ji-Ho Kim, Tae-Seok Ahn

**Affiliations:** Department of Environmental Science, Kangwon National University, Chuncheon 200-701, Republic of Korea

## Abstract

After cut off of inflowing water, Lake Paro, an oligomesotrophic lake lost littoral zone, an important region for the aquatic ecosystem. For the first step of restoration, the artificial vegetation island was installed. The concentration of nutrients in lake water was not sufficient for the growth of macrophyte as total phosphate was ranged from 58 to 83 **μ**g L^−1^. In order to overcome this problem, the hydrophobic substratum for bacterial attachment was selected as buoyant mat material of the artificial vegetation island. In this medium, total phosphate and total nitrogen were ranged from 190 to 1,060 **μ**g L^−1^ and from 4.9 to 9.1 mg L^−1^, respectively. These concentrations were high enough for macrophytes growth. After launching 1,800 m^2^ of AVI in Lake Paro, the macrophytes, *Iris pseudoacorus* and *Iris ensata*, grew well after five years of launching without the addition of fertilizer. Furthermore, fishes were plentiful under the artificial vegetation island, and ducks were observed on the artificial vegetation island. Bacteria using sunlight as energy source and self-designed ecotechnology can be used as an alternative method for the restoration of disturbed littoral zone in oligo-mesotrophic lakes.

## 1. Introduction

**Figure 1:**
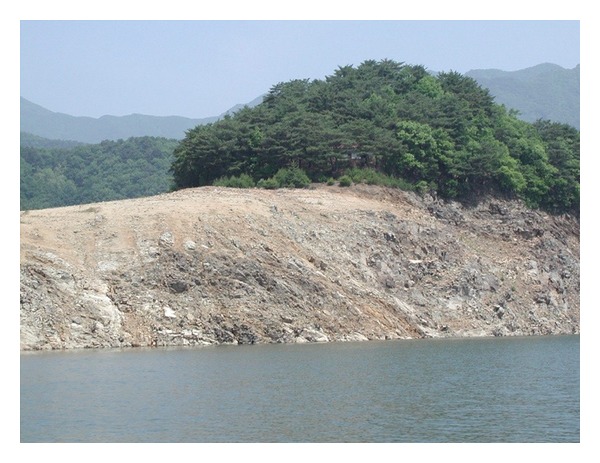
Degraded littoral zone in Lake Paro, Korea. Water level was lowered for lake construction (August, 2002).

Following construction of the Keumgangsan Dam in Democratic People's Republic of Korea in 2001, about 60% of main inflow into Lake Paro was cut off. To protect from possible breakage of the Keumgangsan Dam, a defensive dam, named Peace Dam, was constructed in 2005 near the upper part of Lake Paro. The water level in Lake Paro was lowered from 181 m to 150 m following the construction of the dam (Figure 1). After the decline in water level, this oligomesotrophic Lake Paro experienced problems related to fish habitat such as degradation of the littoral zone and lose of spawning and refuge areas. These problems resulted in a reduction in fish populations such as the common carp (*Cyprinus carpio*) and mandarin fish (*Siniperca scherzeri*) [1].

Restoration of littoral habitat in Lake Paro is vital to promoting the sustainability of the Lake Paro ecosystem. However, the littoral zone at Lake Paro cannot be naturally revegetated easily because of these steep slopes and frequent fluctuations in water level. The emergent macrophytes in the littoral zone are affected by local conditions such as water logged soils, partial submergence and nutrient availability [2]. In some cases, natural vegetation islands comprised of submerged, and emergent macrophytes can deteriorate the fishery and lake water quality [3]. 

Artificial vegetation islands (AVIs) can be ameliorate some of the problems associated with degraded littoral zones where is seriously degraded such as in Lake Paro [4, 5]. The broad concept behind AVIs is to mimic natural floating islands which are common in some lakes across the world. AVIs often consist of macrophytes and buoyant material. AVIs should include adequate biomass in the rhizosphere zone because of the important role of providing habitat for fish spawning [6] and refuge areas for zooplankton [7, 8] and invertebrates [9]. Specially, the effective design of the AVIs in oligo-mesotrophic lakes must include the suitable materials to allow for the accumulation of nutrient supply for macrophytes growth in the AVI, and the biotic components of the AVI are essential [10]. Besides, the materials of the AVI have to attach and support bacteria.

Bacteria play an important role in the accumulation of nutrients [11], and aggregated or attached bacteria have been shown to have an especially effective role in biofilm formation [12]. The bacteria in biofilms are embedded in exopolysaccharides [13], and form microcolonies on hydrophobic substances [14].

Thus, the material used in AVIs in oilgomesotrophic lakes should has a role not only sustaining macrophytes but also supporting an active biofilm community. 

As an alternative littoral zone, we installed the AVI in Lake Paro and observed the growth of macrophytes and ecological changes.

## 2. Materials and Methods

### 2.1. Buoyant Mat Material

According to previous research [15], rubberized coconut fiber was selected for buoyant mat material. After 1 month of submerging the mat made of rubberized coconut fiber into distilled water, we did not find evidence of significant release of nutrients from the mat (data not shown). Before installing the AVI, in order to confirm the accumulation of nitrogen and phosphorus in the mat, three pieces of rubberized coconut fiber (1 m by 1 m at 0.1 m depth) were submerged in the center of Lake Paro for 1 week. After retrieving the mats, the interstitial water was sampled by gravity and analyzed for total nitrogen (TN) and total phosphorus (TP) concentrations using standard methods [16]. 

### 2.2. Installation of the Artificial Vegetation Island

**Figure 2:**
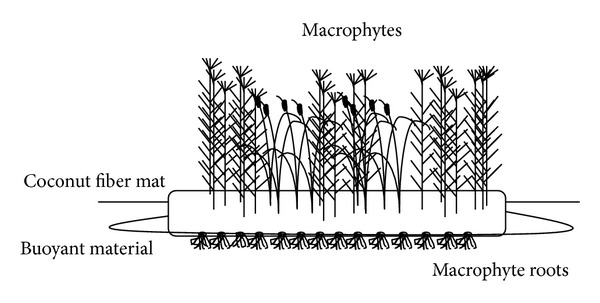
Diagram of AVI launched at Lake Paro. 1,800 m^2^ of AVI was installed on August 2003.

After the effectiveness of buoyant mat material as a nutrient concentrator was confirmed, 1,800 m^2^ of AVI was installed in Lake Paro (38°06′51′′N, 127°49′25′′E) (Figure 2). Two species of macrophytes, *Iris pseudoacorus* and *Iris ensata*, were planted at a density of 9 shoots m^−2^ in the AVI during August 2003. Ten shoots of each macrophytes species were randomly collected and the total length and root length were measured in the laboratory during May 2004. 

### 2.3. Samples Preparation and Water Chemistry

Interstitial water samples from AVI were extracted using sterilized syringes, and lake water samples were collected aseptically with 1L plastic bottle as a control. These samples were transported in cold condition (4°C) and immediately analyzed. 

Total nitrogen (TN) and total phosphate (TP) were analyzed by using standards methods [16]. Measurements were conducted in triplicate. 

### 2.4. Bacterial Abundance

For the direct counting of bacterial abundance, samples were filtered through black polycarbonate membrane filter (Nucleopore, pore size 0.2 *μ*m, dia 25 mm) and stained with acridine orange (100 *μ*L of 1 g/L aqueous stock solution of acridine orange) [17]. Bacterial abundance was counted with an epifluorescent microscope (Olympus BX60, Japan).

## 3. Results 

### 3.1. Nutrient Accumulation in the Buoyant Mat Material

After one week of submergence in Lake Paro, the TN and TP concentrations in the interstitial water were much higher than initial concentrations. Interstitial water samples collected by gravity force and squeezing had TP concentrations ranging from 0.4 mg L^−1^ to 5.1 mg L^−1^ and TN concentrations from 38.5 mg L^−1^ to 489.0 mg L^−1^. These concentrations of collected samples by gravity force were about 100 times and 20 times higher than the concentrations in the lake water, respectively. Water samples collected by squeezing water had approximately 1,200 times the lake water column concentrations of TP and 50 times higher than lake water column TN concentrations (Table 1). 

### 3.2. TP and TN in the AVI and Lake Water

**Figure 3:**
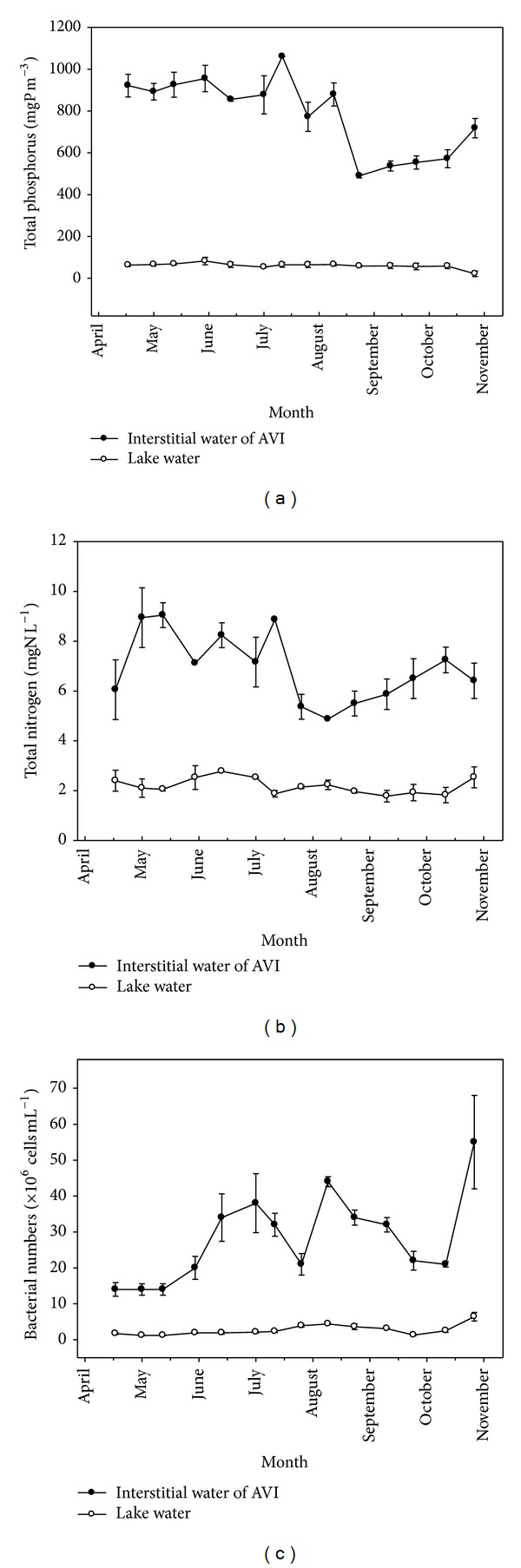
Variations in TP (a), TN (b), and bacterial numbers (c) in AVI interstitial water and ambient lake water in Lake Paro in 2004.

Variations in interstitial TP concentrations in the AVI and lake water are shown in Figure 3(a). During the macrophytes growing period, TP concentration in lake water and interstitial water of AVI ranged from 53 to 83 *μ*g L^−1^ and from 490 to 1,060 *μ*g L^−1^, respectively. TP concentrations in the AVI were 17 times higher than those in lake water. TN concentrations in the AVI interstitial water ranged from 4.9 to 9.1 mg L^−1^, approximately 3.5 times higher than those of lake water (Figure 3(b)). 

### 3.3. Bacterial Abundance and Macrophytes Growth

Variations in total bacterial numbers in the interstitial water of AVI and the lake water are shown in Figure 3(c). In interstitial water of AVI and the lake water, the bacteria abundance ranged from 1.4 × 10^7^ to 5.6 × 10^7^ cells mL^−1^ and from 1.2 × 10^6^ to 6.5 × 10^6^ cells mL^−1^, respectively. Bacterial abundance in the interstitial water was always higher than the bacterial abundance in lake water, about 5 to 18 times higher. 

**Figure 4:**
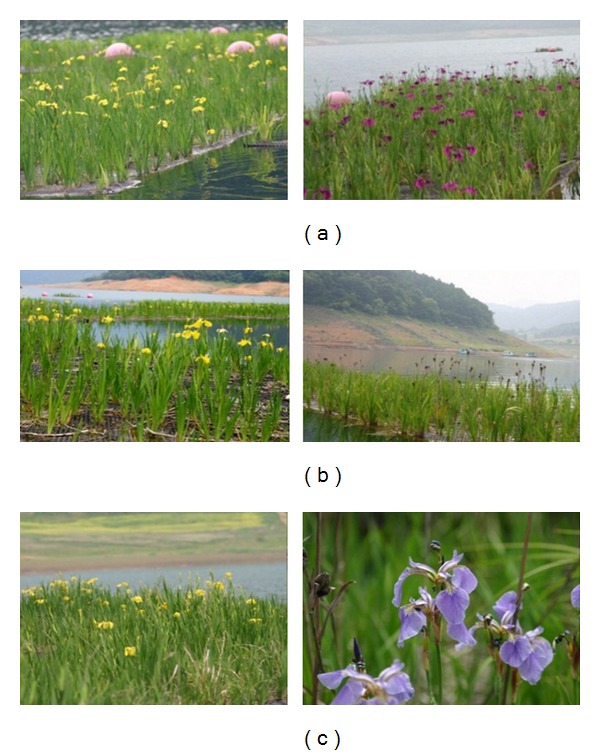
Iris flowering on AVI in Lake Paro (*Iris pseudoacorus* (left), *Iris ensata* (right), and May, 2004 (a), June, 2005 (b), May, 2008 (c)).

Immediately following the launching of the AVI in Lake Paro, macrophyte growth was prolific. During late summer and autumn the macrophytes grew well and in the spring of 2004, healthy new shoots were produced by the 1-year-old macrophytes in the AVI (Figure 4). The length of roots of both macrophytes ranged from 50 to 100 cm, and the lengths of leaves ranged from 60–70 cm. 

## 4. Discussion

The artificial vegetation island (AVI) in Lake Paro can be regarded as unique wetland ecosystem installed within an oligomesotrophic lake. AVIs can be effective for wetland restoration in places where the littoral zone has been severely degraded. Similar to natural floating islands, nutrient concentrations within the AVI are often higher than the ambient lake water (control) [18]. 

In Lake Paro, the AVI installation was selected with a focus on the macrophyte community as a key functional role [19]. The Macrophytes absorb inorganic and organic nutrients, provide habitat for beneficial microorganisms, and promote the breakdown of organic matter by oxidation in the root system [20]. 

In the AVI, the central component of the macrophytes community is the submerged root system. Some macrophyte roots spread through the mat material (coconut rubberized fiber) to obtain nutrients, and other roots hang directly in the water column. These roots showed the proliferation of bacteria. In our study, we did not analyze the physiological functions of the macrophyte roots, but we assume that their physiological and ecological functions are similar to the functions of submerged macrophytes, which include providing refuge areas for pelagic zooplankton [21], for eggs of fishes [6] and for invertebrates [8]. 

The AVI is composed of artificial buoyant material. In eutrophic and shallow lakes, the mat material may not be important for macrophytes growth, because the nutrients for macrophytes growth are supplied from the lake water or sediment. However, in oligotrophic lakes, the mat material might play a role of nutrient supply. We carried out the preliminary experiment for evaluating the different mat materials, with hydrophobic rubberized coconut fiber selected as most suitable [15]. 

The surface of rubberized coconut fiber is hydrophobic, so this substratum of the AVI can be used for bacterial adhesion. Desorption was considerably greater on the hydrophilic substratum, whereas desorption from the hydrophobic media was almost negligible [22]. Moreover, bacterial adhesion to the hydrophobic surfaces appears to be rapid, and binding may be stronger than those on hydrophilic surfaces [23]. Therefore, interstitial water of rubberized coconut fiber mat contains high concentration of nutrients, even though the nutrient concentrations in the lake water are low.

We did not measure the biomass of macrophytes, *Iris pseudoacorus* and *Iris ensata*. It is likely a large portion of nitrogen, and phosphorus accumulated by bacteria could be transported into the macrophytes biomass. The phosphorus and nitrogen contents of *Iris*, grown at nutrients rich pond, have been measured to be 0.2% and 2.4%, respectively. Assuming a productivity of 5 g dry weight m^−2^ day^−1^, a daily uptake of 0.01 g P m^−2^ and 0.12 g N m^−2^ can be estimated [24]. Direct uptake of nutrients by macrophytes from bulk lake water might be limited, because of the low nutrient concentrations. Therefore, the nutrients for macrophytes growth are from the mat. This hypothesis is supported by the healthy appearance of macrophytes leaves and active flowering, suggesting that the nutrient supply from the mat was sufficient [25]. 

The bacterial abundances at AVI water were about 50 times higher, and the bacteria cell size was larger than those found in ambient lake water. This indicates that the AVI supports a newly created bacterial ecosystem which is different from the ambient lake water ecosystem. 

Another ecological function of the AVI is supporting a new biotope. We observed birds and fishes thriving after the installation of the AVI, and one study showed that an AVI consisting of *Phragmites japonica* and other macrophytes supported 43 species of insects after 2 years of installation [5]. 

In this study, we did not measure the diffusion of nutrients from AVI to lake water. The higher nutrients concentrations in the AVI interstitial water result from dilution, and a detailed nutrient budget would be needed to quantify all the processes involved in nutrient release and retention in the AVI. This could be the focus of future research. 

## 5. Conclusion 

**Figure 5:**
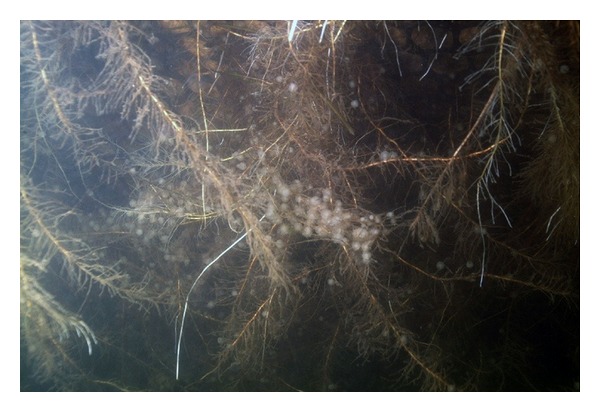
Fish eggs attached to macrophyte roots under the AVI (on June, 2007).

This study focused on restoring the functions of the littoral zone in an oligomesotrophic lake by installing an AVI. Lake Paro is an oligomesotrophic lake with insufficient nutrient concentrations to support vigorous growth of macrophytes. However, any addition of fertilizer to the AVI in Lake Paro is not desired and is prohibited. We found that following the AVI installation, the bacteria community in the lake adhere to the hydrophobic substratum. Biofilms bacteria accumulated TP and TN from the lake water. The roots of macrophytes beneath the AVI provided refuge areas for eggs of fishes (Figure 5), zooplankton, and invertebrates and consequently attracted planktivorous and piscivorous fishes. Birds were also attracted to the AVI. The AVI macrophytes community has continued to grow without any special treatment; therefore, this system may permanently function only if there is no mechanical damage. 

## Figures and Tables

**Table 1:** TP and TN concentrations in interstitial water of buoyant mat material after 7 days of submergence in lake water (May, 2003).

	T-P (mg P L^−1^)	T-N (mg N L^−1^)
Lake water	0.004	1.9
Interstitial water by gravity force	0.4	38.5
Interstitial water by squeezing	5.1	489.0
